# Why Tenure Responsive Land-Use Planning Matters: Insights for Land Use Consolidation for Food Security in Rwanda

**DOI:** 10.3390/ijerph16081354

**Published:** 2019-04-15

**Authors:** Uchendu Eugene Chigbu, Pierre Damien Ntihinyurwa, Walter Timo de Vries, Edith Ishimwe Ngenzi

**Affiliations:** 1 Chair of Land Management, Technical University of Munich, Arcisstrasse 21, 80333 Munich, Germany; pdamien.ntihinyurwa@tum.de (P.D.N.); wt.de-vries@tum.de (W.T.d.V.); 2 Department of Land Administration and Management, Institut D’enseignement Supérieur de Ruhengeri, Musanze Street, Ruhengeri NM 155, Rwanda; ishimweedith@gmail.com

**Keywords:** food security, land, land use consolidation, land-use planning, land tenure, rural development, Rwanda, tenure responsive, tenure responsive land-use planning, tenure security

## Abstract

Land use consolidation aims to address food insecurity challenges in Rwanda. However, there is contradictory evidence on whether this tool has met food security objectives or not. This study addresses two questions: How has the land use consolidation improved (or not improved) food security at the local level? How can food security challenges be addressed using a renewed approach to land use consolidation that adopts a tenure responsive land use planning procedure? We investigate these questions in Nyange Sector (in the Musanze District) of Rwanda using mixed research methods. The study generates theoretical and policy relevant outcomes. Theoretically, it links the concept of tenure responsive land-use planning to food security improvements. Policy wise, it provides an operational framework for implementing land use consolidation to make it more responsive to food security (based on tenure responsive land-use planning measures) in Rwanda.

## 1. Introduction

In most sub-Saharan African (SSA) societies, land use practices are rooted in ensuring improved livelihood options, community cohesion and food security [1]. Even though various approaches are being applied towards achieving this aim—both in scientific and cultural terms—the region continues to face various land-related challenges. Some of these challenges include land degradation [2], water pollution [3] rural and urban poverty [4], poor environmental sanitation, climate change related problems [5], gender inequality (especially women’s lack of access to land) [6], inefficient land administration [7], loss of culture [1], land fragmentation [8], to mention a few. Out of the many problems bedeviling SSA countries, two critical challenges the region faces are how to deal with the widespread development of urban areas [9] and how to improve livelihoods development of the rural areas in a “responsible” manner [10,11]. Compounding the severity of these problems are the persistent threats posed by climate change, poverty, disaster risk, and lack of access to land (and other natural resources) for use for different aspects of human development. This study takes a rural dimension to investigate one of these problems. 

The challenge of improving food security (or food insecurity as a treat to improved rural livelihoods) is increasingly understood to have a relationship to the types of vulnerabilities its users face on land, as well as to the type of privileges (and rights) owners (and users) enjoy on land [12,13]. For example, reduced food security could be caused by environmental degradation, poor energy usage, natural disasters or the way people use land [14,15,16,17]. Also, the level of tenure security land users and landowners have on a parcel of land can influence its productivity in both positive and negative ways [8,18,19,20]. One of the many countries in Africa where the relationship between land use and food security (among many other land-related problems) has become a policy concern is Rwanda. The land challenge in Rwanda is a complex one despite that the country has surveyed, adjudicated and registered all its land. The country-wide registration of land in Rwanda has led many scholars to assume that Rwandan rural landowners have high land tenure security that will enable them to participate fully in the development process of the country [6,7,10,18,21]. This assumption is however not completely valid due to a number of additional land related problems. First of all, Rwanda is the most densely populated country in Africa. Its dense population is one of the factors that makes land a critical resource in the country. The majority of the country’s working population earn their livelihood from land (especially through agriculture) and related employment [21,22]. Rwanda’s food security challenges have been blamed on the scarcity of land, and land policy related strategies (that are not adequately responding to the nature of challenges being faced by the country) [23]. Secondly, land tenure practices aside, the unique topography and high population densities of Rwanda are some of the reasons the country has a highly fragmented landscape which is in dire need of consolidation [8,24,25,26,27,28]. The consequence of these particular characteristics is that land use decisions remain contested and that food insecurity persists. 

In recognition of the persistence of food insecurity in the country—and from the rural perspective—the government has made policy efforts towards improving the situation. The government has put improved agricultural production and food self-sufficiency at the top of its development agenda [29,30]. It introduced land use consolidation (LUC) measures since 2008 as a component of the Crop Intensification Program (CIP) to broadly meet food security by boosting the national agricultural production. However, the outcome of LUC remains questionable and debatable. There is contradictory evidence from literature on whether the program has met food security objectives or not. On the positive side, some studies (especially Rwandan government reports) claim that LUC has doubled (and even tripled) agriculture yields of the priority crops, and thereby increased food availability and food quantity at the national level [24,31,32,33,34,35]. On the negative side, Pritchard [29] argues that due to the rapid and forceful implementation of the program, “tenure and agricultural policies are unnecessarily undermining the livelihood stability of rural subsistence farmers”. Brown and Hughes [23], note that “land use consolidation and limitations on land subdivisions have produced emerging threats to tenure security”, as well as food security. Others argue that the program leads to monoculture farming since in its implementation all farmers with closed parcels are asked to grow only eight priority food crops [8,24,26,31,32,33,34]. This position is emphasized by the Rwanda National Food and Nutrition Plan 2013–2018 which recognizes that despite significant economic and poverty reduction progress brought by different governmental programs including LUC regarding tremendous increases in national agriculture production of the priority crops, improvements in nutrition and household food security remains a foundational issue [36,37] Irrespective of what side of the argument these scholars stand, they all agree that there are still worrying levels of inappropriate land use practices and livelihood options that cause food insecurity at the national level [2,24,26,30,31,32,33,34,35,36,37,38,39,40]. 

This study deals with an important question in Rwanda’s land policy and land reform implementation at the local level. It takes a critical look at the impact of the LUC program on food security at the local level. It uses the FAO Food Security definition-oriented approach (which puts focus on the households and individual’s dietary needs and food preferences) to assess food security status and driving factors before and after the LUC in the rural sector of Nyange, in Rwanda. This is done with the objective of discerning a renewed approach of LUC through tenure responsive land-use planning. The study approaches this objective by answering two key question that focus on the extent to which LUC impacts food security and what should be the recommendations to increase the effectiveness of LUC towards food security. The questions are:

- How (much) has the program improved (or not improved food security) in at the local level local in Rwanda?
- How can food security challenges be addressed using a renewed approach to land use consolidation that adopts tenure responsive land use planning procedure.

By answering these “how-to” questions, this study departs from existing literature in two ways. Firstly, it answers the question of whether LUC has an impact on food security at the local level. Considering that many of the contradictory literature (arguing for and against) on the food security performance of LUC at the national level, investigating the impact of LUC at the local level is necessary because it is possible that LUC may not have improved food security nationally but may have had positive impacts in pockets of administrative sectors in the country. Secondly, by focusing on how a tenure responsive land-use planning approach can be used to make LUC respond to the food security needs of the people, the study introduces a new approach to food security improvements. 

In order to answer these questions, the study begins by framing the tenure responsive land-use planning as the conceptual basis for ensuring food security improvement at the local level. This is followed by description of the methodology, outcomes and discussion leading to a proposal for making Rwanda’s LUC to generate food security through a responsive land-use planning approach. Finally, the study concludes.

## 2. Literature Review

### 2.1. Framing Tenure Responsive Land-Use Planning in Response to Food Security: Delineation of Intertwined Concepts, Definitions and Relationships 

**Figure 1:**
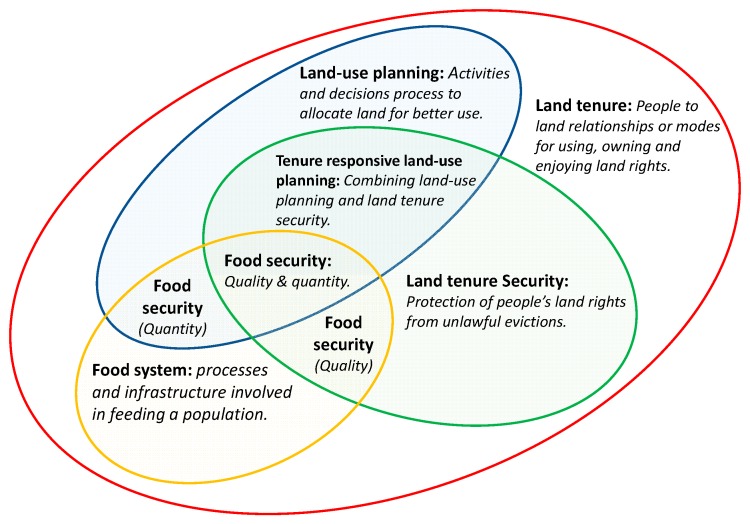
Conceptual delineation of key terms used in the study.

To understand tenure responsive land-use planning in response to food security, six major terms are worth understanding. They are land-use planning, tenure responsive land-use planning, land tenure, land tenure security, food systems and food security. All of these concepts share a relationship with land. Individually and collectively, they also have causal relationships with food security. Although the focus of this study is on tenure responsive land-use planning and food security, it is important to grasp the meanings of these concepts and the general relationships they share. This is important for understanding the relationship between tenure responsive land-use planning and food security (Figure 1).

Land tenure is an overarching concept that entails the manner in which people use own and enjoy rights on land (including privileges, obligations, restrictions and responsibilities). It reflects the relationship people, communities, groups and individuals share with land [17,18,23,24,25,41,42,43,44,45,46,47,48,49,50,51,52,53,54]. Land tenure and land tenure security are two interwoven concepts. On the one hand, land tenure means the “manner in which the rights, restrictions, and responsibilities that people have on the land (and property) are held” [13,55,56]. Land tenure is that which defines and shapes the way people own, hold, and enjoy rights to land. Hence, it embeds local realities (regarding people-to-land relationship) in regard to legal, social and cultural practices relating to land. On another hand, tenure security implies the “rights individuals and groups have to effective protection by the state against forced eviction. Under international law, this entails permanent or temporary removal against the will of persons, families and communities from the homes and land that they occupy, without the provision of, and access to, appropriate forms of legal or other protection” [18]. When land tenure is secure, individuals (and groups) enjoy the ownership, use, and exercise of various rights on land with higher freedom. Tenure security manifests in de facto or de jure ways, and it can be established through legal or social agreement or recognition [10,12,13,17,41,42,43,44]. So, it can enable or sustain access to and availability to land (especially when consciously planned to achieve these) for a specific period (or in perpetuity). Moreover, by doing this, it has the potential to enable and sustain access to and availability of food (thereby making food-intake and its stability possible) so far as the individuals involved have the requisite knowledge about nutrition, food governance and issues related to laws and regulations are appropriately conducted [57,58,59,60]. In the context of land tenure (considering that food can also be made available via imports and food aid), the availability of food as may be determined by tenure security connotes the quantity of food of acceptable quality possible through food crop production. Access to food implies that individuals and groups can acquire the available food resources for use. Food usage entails that the acquired food is used for balanced nutritional intake. Food sustainability is about having the necessary stability to ensure that access, availability, and use of food are stable enough to ensure a normal state of living. As food production (and its security) is dependent on the access, availability, appropriate use and sustainability of land (regarding access, availability, use, and sustainable management), adequate management of land (through land-use planning) has a direct impact on food security. What makes this relationship possible is tenure security on land. Land tenure exist in any place where there is human to land relationships (so it exists everywhere). However, land-use planning and land tenure security do not exist everywhere except where they have been consciously conditioned to exist.

Land-use planning and tenure responsive land-use planning are two other entangled concepts. Land-use planning, unlike tenure responsive land-use planning, is an old concept in planning literature. Myriad of definitions exist on the concepts of land-use planning from disciplinary and policy angles. However it is defined, land-use planning usually alludes to “activities and decisions concerned with guiding the allocation and use of land in patterns that enable improvements in peoples’ way of living” [41]. It is a relevant land intervention measure for development at the local level because “it is one of the most sensitive political issues in any country since it affects people’s livelihoods and the essential needs of communities” [12,13,17,42,43,44]. However, tenure responsive land-use planning is an integrative concept that posits to combine land-use planning and land tenure security (otherwise referred to as tenure security). Just any of the other concept defined in this study, land-use planning is done within an environment of land tenure but not necessarily in an environment that strives to include tenure security. Tenure responsive land-use planning is done inclusive of tenure security. Land tenure security and land-use planning were two separate areas of research until the works of the Global Land Tool Network (of UN-Habitat) which led to the seminal works of Chigbu et al. [10,12,13,17,42,43,44]. In their works, they [10,12,13,17,42,43,44] argued for a combination of tenure security and land-use planning concepts (and practice), leading to the coinage of the term, tenure responsive land-use planning. The concept of tenure responsive land-use planning recognizes that “separately, land-use planning and tenure security improvements remain the two strongest forces for influencing spatial transformations and development” and combined they “play crucial roles in achieving development objectives” [13]. Land-use planning assures appropriate use of land while tenure security is the precondition for sustaining livelihoods through sustainable agriculture, among others. This makes its combination with tenure security imperative for food security improvement. Tenure responsive land-use planning is a specific type of land-use planning process that can wholly or incrementally establish land tenure security [12,13]. By incrementally or wholly ensuring land tenure security, it can generate elements of food security when consciously implemented with food security as one of its key objectives. 

Food systems and food security are intertwined concepts. A food system includes the various processes (including infrastructure) that involve making food available (in quality or quantity or both) to feeding a population [60,61,62,63,64,65,66,67,68,69,70,71]. The process within a food system could be land policy and agricultural policy making and implementations for ensuring the eradication of hunger. A food system can interact with other land related processes or conditions (such as land-use planning and land tenure security) to produce food availability (in terms of in quantity or quality. This study views food security as a situation where people have physical and socioeconomic access to sufficient nutritious and safe food that meets their dietary needs (and food preferences) for an active and healthy life [45,46,47]. This definition of food security captures four critical components of food security, which include food availability, food access, food usage, and stability to continue to enjoy food usage as a healthy way of life [48,49,50,51,52,53]. These four components can also serve as possible indicators for measuring (quantitatively) or evaluating (qualitatively) food security at national, regional, and at the local levels (household and individual levels). Whenever one of these four aspects are not fulfilled or met, people may suffer from hidden hunger. A food system has the potential to activate and enable land interventions (or development measures) to produce food security outcomes. 

### 2.2. Tenure Responsive Land-Use Planning as a Generator of Food Security: A Framework

**Figure 2:**
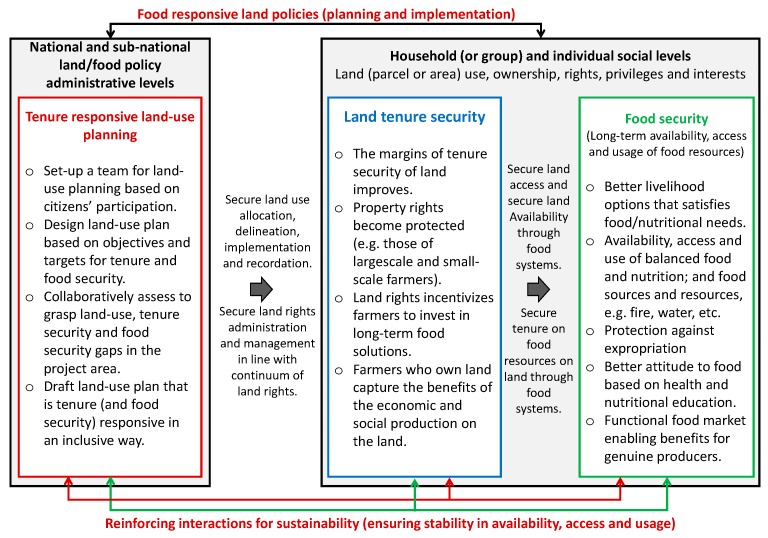
Framework for food security through tenure responsive land-use planning.

The major link between the operationalization of tenure responsive land-use planning and the outcome of food security policies is tenure security. A variation in tenure security influences the outcomes of food security policies when tenure security actors interact with food systems [8,12,29,30,42,43,57,58,60,61,62,63,65,67,68,69,70,71]. This link is of primary interest in framing any approaches to improving food security through tenure responsive land-use planning. It is also necessary for analyzing, and designing future (and improving existing) land-use decision-making) processes to ensure that they respond to food security needs of their stakeholders. That is why this study frames food security as a possible outcome of tenure responsive land-use planning (Figure 2).

Land-use planning, especially in rural areas, usually involve the participation of farmers who own land or the involvement of farmlands owned by people eager to improve their food security situation. In such a situation, the operational framework ensures that such farmers or land users can capture the benefits of expected socioeconomic production on the land to improve their food security. However, this demands a land-use planning process that allows securing tenure and property rights. It calls for a process that allows for, at least three vital systematic aspects: a tenure responsive land-use planning, a land tenure security, and a food security component (Figure 2). The tenure responsive land-use planning component is embedded in policymaking. There are effects in both land tenure security and in food security. 

The tenure-responsive component when guided by land (food responsible) policies provides guidance at administrative implementation levels and also enables the sustainability of any outcomes (in this case, food security). Where this is the case (and there is need to do land-use planning), as a starting point will be to set up a team of participants based on the principles of citizens’ participation and continuum of tenure (and land rights). Participation will ensure that everyone’s rights to engagement are ensured [13]. A continuum of land rights approach implies that participants (and administrators) must recognize that “different types or levels of tenure security may prevail in the everyday lives of people because of the social rules, customary practices and laws within a particular land jurisdiction” [13]. So, land-use planning has “to be implemented with the recognition that land rights are not static, but are manifested in various forms across a continuum of rights–tenure security can be enhanced through the recognition of individual, community and indigenous tenure rights by creating a better understanding of social and legal protections for various means of land tenure” [13]. Taking these steps is not possible unless (in the design of a land-use plan) tenure security objectives and targets are adopted. This implies collaboratively assessing situations to grasp land-use, tenure security, and food security gaps in the project area; and then drafting a new land-use plan that is tenure (and food security) responsive in an inclusive way. Inclusiveness is necessary to ensure access to land to unlock household insecurity by women and other underprivileged groups. This is important to enable land users and farmers who own land to capture the expected socioeconomic benefits from their land. 

The second component is influenced by policy (hence, it is an effect susceptible to policy effect). This component can be influenced by secure land use allocation, delineation, implementation and recordation; and secure land rights administration and management in line with a continuum of land rights. It has two parts–the land tenure security and food security–with the former influencing the later through secure availability and access to land; and secure tenure on food resources on land. Where the margins of tenure security of land improve, property rights become protected (e.g., those of large-scale and small-scale farmers), then land rights incentivize farmers to invest in long-term food solutions, and farmers who own land capture the benefits of the economic and social production on the land. This will have a positive effect on food security it will lead to better livelihood options that satisfy food/nutritional needs, protection against expropriation, better attitude to food based on health and nutritional education and a functional food market enabling benefits for genuine producers. This will ultimately result in the availability, access, and use of balanced food and nutrition (and including land related natural resources linked to natural food sources for energy and water access).

The logic behind the framework is that tenure responsive land-use planning ensures protection of property rights to land users (e.g., small-scale farmers) and enables them to take critical steps to invest in long-term solutions that gives them access (and makes land and food available) for use in their quest to fight poverty and hunger. This has been found to be right in Zambia [72], Tanzania [54], Namibia, Laos, Philippines, Brazil, Chile, Ghana and Ethiopia [12,17,73,74]. It, therefore, makes sense to assume that this is possible in a country like Rwanda. This study, therefore, puts focus on the LUC being practiced in Rwanda –with the aim of reaching an improved food security implementation strategy through tenure responsive land-use planning. 

## 3. Materials and Methods

**Figure 3:**
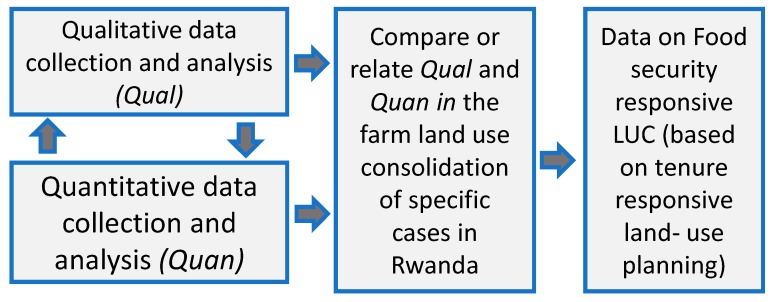
Convergent parallel mixed methods research design.

This study follows a mixed methods approach (Qualitative and Quantitative) and a pragmatic world view which fits for a better understanding of complex problematical situations and the consequences of actions. Food security is a very complicated variable which cannot be easily identified by one predetermined method, while the evaluation of consequences of LUC as an action to overcome it similarly needs a multidimensional approach. Creswell [75,76] note that this kind of approach gives the chance to rely on different methodologies and convergent data collection and analysis techniques which gives a better understanding of the problem and enables better solutions. This dictated the use of both qualitative and quantitative methods based on data collected simultaneously from April 2017 to June 2017 as shown in Figure 3. 

The data collection relied on a household survey (using semi-structured questionnaires) based on semi-structured face-to-face interview. The survey focused on discerning the values of food security indicators, the perceived driving factors for what influenced food security for individual farmers, their observed impacts of LUC implementation strategies on food security, the type and variations in how actors contributed to the LUC implementation process, and the level of farmer’s satisfaction with the LUC program. Field observations of agricultural fields and reviews of food security policy document aided in validating and triangulating the responses of farmers and linking the specific fields to known and documented agro-ecological conditions of the Nyange sector

### 3.1. Site Selection

The data collection took place in the Nyange Sector of the Musanze District (Northern Province of Rwanda). The choice for this study area was purposively driven by the fact that this Sector is one of the project areas where the LUC program is facing post-implementation resistance from farmers (particularly against the priority crops of the government) in the Northern Province of Rwanda. It is important to mention that the resistance of farmers against LUC in the area cannot be linked with its failure to meet food security, as it is a post-implementation resistance. The resistance is considered as an outcome of the failure of LUC to meet its food security targets, rather than a cause or one of the factors. The farmers in this area have been complaining that the Governments priority crops are unsuitable to their local agro-ecological conditions and less economically valuable than the non-priority crop grown in the area before the introduction of LUC [8,23,24].

**Figure 4:**
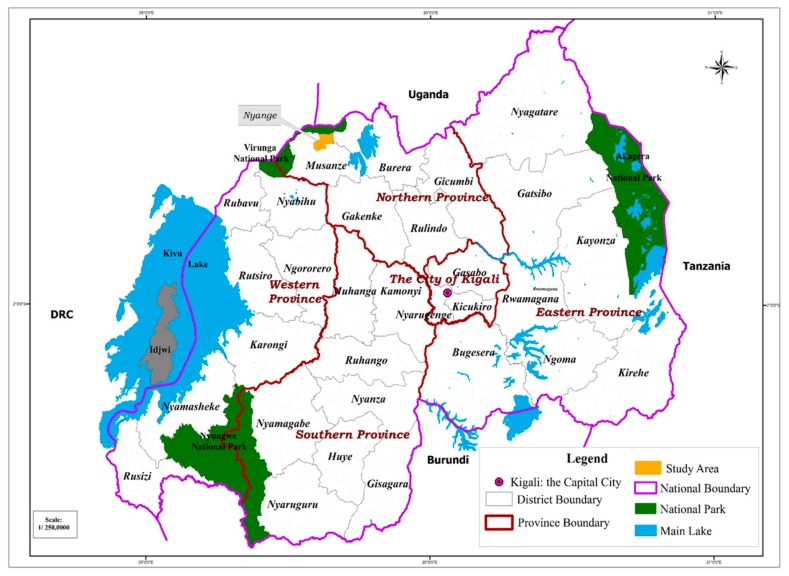
Nyange sector (case study) location in Rwanda.

Furthermore, the Nyange Sector, as well as the whole Musanze district is a volcanic region. It has experienced changes in weather conditions and rainfall patterns which have led to severe floods as natural risks or disasters often associated with the destruction of food crops and total loss of agricultural production with food insecurity as an obvious consequence over time. Figure 4 shows the location of the case study.

### 3.2. Data Collection and Anylsis Methods

The data collection was done in five cells (*Kabeza I, Kivugiza I, Ninda, Muhabura, and Kamwumba*) of Nyange sector in Musanze district. The choice of all the five cells was not for the comparison purpose, but for validity and reliability purposes. A combination of stratified random sampling and purposive sampling methods were used for the selection of respondents. In total, 72 respondents representing more than 15% of the total number of households in the sector were randomly selected for the household survey using the formula of Lykken et al. [77] which is appropriate for very homogeneous populations, and six key respondents purposively selected (five cell agronomists, one sector agronomist) respectively were interviewed for primary data collection. Only one household representative member was chosen to respond to a set of questions during the household survey. The choice of the six key informants was motivated by their critical roles in the implementation of LUC and CIP programs at the local level and their likelihood to bear the information about food security in the area as agronomists. The questionnaires were drawn in English language and translated in Kinyarwanda. All the questions were asked in Kinyarwanda as the local language and the answers translated in English by the authors later. This technique of combining households survey (quantitative non-experimental) and other qualitative methods in the assessment of programs and policy impacts is very famous in social sciences and has been widely used by many other researchers in the same context [8,20,24,26,33,34,35] which makes it a more credible and reliable approach. The Triangulation and Back-checking techniques were used to check the validity and reliability of the collected data, and were chosen for their extensive use in mixed studies like this one.

The information about the failure of LUC to meet food security in the study area was primarily obtained by collecting the perceptions of respondents about the status of household food security before and after the introduction of LUC from the household representatives (Heads) through a household survey and the key informant’s interviews. All the households’ respondents were asked to choose one answer among four different pre-established indicators of food security in their households after the introduction of LUC. These four indicators include, first, optimal uptake of nourishment which indicates food security in all its aspects (food quantity, food quality, food availability, food accessibility, food utilization or usage, and food sustainability). Second, sufficient balanced food availability which indicates the presence of food security in terms of the availability of diverse foodstuffs (food quality) in sufficient quantity. Third, sufficient access to food which indicates the accessibility of food in terms of quality and quantity for food security. Lastly, insufficient balanced food availability which indicates food insecurity in terms of the unavailability of diverse foodstuffs (food quality) and quantity. On the other hand, the key respondents as more educated people, were asked to compare the status of household food security in all its aspects such as food quantity, food quality, food sustainability/stability, food availability, food accessibility, and food usage/utilization before and after the introduction of LUC in their area. Each key respondent was also required to give a motivation for this comparison. The information about the level of farmer’s satisfaction about LUC and the reasons for their satisfaction level has also been gathered through a household survey, in order to check whether food insecurity indicators could be one of the main factors of satisfaction, and which indicators could be linked with the failure of LUC. This helped to answer the questions about whether and how LUC failed to meet food security. Both descriptive statistics along with text description were used to analyze quantitative and qualitative primary data to draw statistical and thematic conclusions about the research questions. A correlation diagram drawn from the primary data, literature review and the authors knowledge about LUC and Food Security nexus was used to show the causal-effects relationships among different factors and variables in different food insecurity scenarios before and after LUC in the study area. This helped to show how LUC failed to meet its food security objectives as a multidimensional concept, rather exacerbated the issue by contributing to other forms of food insecurity scenarios when combined with exogenous factors like climate change and market imperfections among others. In order to verify why and how LUC influences food security targets, the study performed an analysis of the existing LUC policy (objectives and guiding principles) and of its formulation and implementation process in the study area. The main target information was the level of farmer’s participation in the process, which could give insights about the independency of land use rights as an indicator of tenure security and factor of food security, as pre-established in the conceptual framework in the literature section. LUC process and main stakeholders’ diagram was drawn from the information gathered from the literature and governmental reports, along with the primary data from farmers during household survey and key informant’s interviews. The level of farmer’s participation was assessed by asking the household members during the household survey whether they participated in 3 main activities of LUC implementation process (provision of parcels and farming activities, choice of the priority crop suitable to their area, and the post harvesting activities). Once the Gap found, it has been correlated/associated with Tenure Responsive Land Use Planning Principles in order to propose a better renewed approach to LUC to make it more food security responsive as solution to the main study problem. A deductive review of the literature about tenure responsive land-use planning principles, LUC guiding principles and Food Security aspects and components was performed to identify the gap in their relationships in the study area, answer the question about the reasons for their failure, and propose new solutions to mitigate food security challenges through tenure responsive land-use planning as secondary data sources to supplement the primary ones.

## 4. Outcomes and Discussion: Has Land Use Consolidation Improved Food Security? If Not, How and Why?

### 4.1. Farmers’ Perceptions Show That the Outcome of LUC Did Not Support Household Food Security

**Figure 5:**
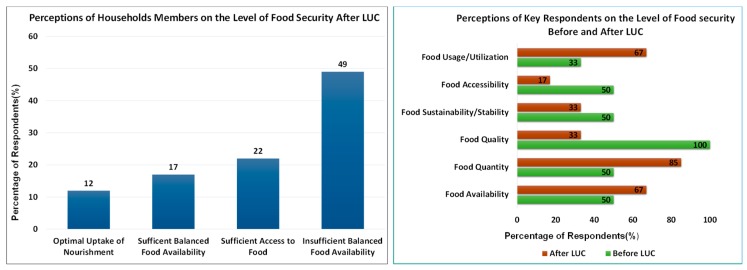
Scenarios of food security in Nyange Sector.

The study compared the before-and-after situations of the LUC to grasp its food security impacts. The level of food security was determined from the perception of critical informants and household members about it at the household levels (see Figure 5), along with the existing reports about food security status before and after the introduction of LUC.

The majority of household members (49%) report to suffer from insufficient balanced food availability in their daily life after the introduction of LUC, while the majority of the responses from the key informants confirmed the decrease in food quality, food sustainability and food accessibility (at 33%, 33% and 17% of respondents respectively) as indicators of food insecurity at the sector level, despite the increase in, food quantity, food usage/utilization and food availability (at 85%, 67% and 67% respectively). In order to understand why this situation arose, it is necessary to understand the factors that reduced food security in the Nyange Sector. 

The question that arises is how and why has the LUC not been responsive to food security needs of the rural people? What are the gaps that exist between principles and practices? Answering these questions necessitates a critical examination of food security status and its driving factors before and after its introduction, and its process of implementation. This is necessary to understand the reasons of its unresponsiveness to food security, and possible ways to improve it. 

### 4.2. Specific Scenarios Explaining How and Why LUC Failed to Ensure Food

Understanding how the LUC fail to ensure food security in Nyange Sector demands to outline the specific scenarios and drivers of food insecurity before and after the introduction of LUC. Participation in land decisions is mandatory for achieving food security. The reason for this assertion is that a path to achieving tenure security cum food security through LUC will be to strengthen citizens’ involvement by making all activities participatory and inclusive in their operational (and decision making) aspects.

**Figure 6:**
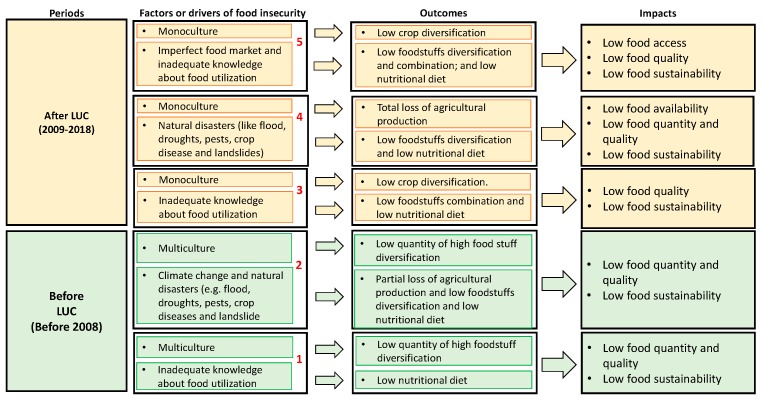
Scenarios showing the failure of LUC to ensure food security in Nyange Sector.

An understanding of the impacts of a specific program on livelihoods needs a careful assessment of the socioeconomic situations of the beneficiaries before and after its introduction. In this study, the views from the key informants and farmers about the level of food security before-and-after LUC alongside a combination of information from the key informants and household survey led to an understanding of the food (in)security status from a before-and-after perspective (Figure 6). 

The above diagram depicts scenarios (with a focus on factors, outcomes, and impacts) of food insecurity before and after the introduction of LUC. The scenarios—*Before LUC (1-2)* and *After LUC (3-5*)—a combination of factors led to low levels of food access, food quantity, food quality and the inability of farmers to sustain themselves with what they have (which are indicators of food insecurity at the household level). The two scenarios (*Before LUC*) and the three scenarios (*After LUC*) show how LUC did not improve food security after its introduction respectively below:

- Before LUC 1: In this situation (before the introduction of LUC), farmers were practicing the subsistence agriculture (based on the combination of multiculture agricultural practices), but there was a presence of inadequate knowledge of food utilization by the farmers. This led to low food quality, quantity, and sustainability as indicators of food insecurity at the household level. This was as a result of low foodstuff diversity combination and low balanced nutritional diet intake.
- Before LUC 2: In this situation (also before the introduction of LUC), farmers were practicing the subsistence agriculture (based on the combination of multiculture agricultural practices). However, due to the effects of climate change (change in rainfall patterns and weather conditions) and natural disasters (floods, pest attacks, and droughts), farming led to low food quantity, low food quality, and low food sustainability (food insecurity). This was mainly due to a partial loss of agriculture production of food crops in case of natural hazards occurrence, low foodstuff diversification, and low balanced nutritional diet intake.
- After LUC 3: Under this situation, LUC’s dependence on monoculture and the farmers’ inadequate knowledge of food utilization lowered food quality intake and sustainability (food insecurity). This was mainly due to low crop diversification (as is always the case with monoculture), low foodstuff combination and low balanced nutritional diet intake.
- After LUC 4: In this situation, LUC’s dependence on monoculture, combined with the effects of climate change (change in rainfall patterns and weather conditions) and natural disasters, (floods, pest attacks and droughts) led to low food availability, low food quality, low food quantity and low food sustainability (which are indicators of food insecurity) through the total loss of agriculture production of priority crop in case of natural hazards occurrence. In this case, LUC acts as a bridge of climate change towards food insecurity.
- After LUC 5: In this situation, LUC’s dependence on monoculture, combined with the presence of imperfect food market and inadequate knowledge about food usage caused low food quality, accessibility, and sustainability (food insecurity). In this case, LUC induces the reduction in agriculture production of priority crops which increases their prices on the market, makes them unaffordable and inaccessible to the poor farmers for foodstuffs diversification and nutritional balance purpose.

It is important to reiterate that the Government of Rwanda introduced LUC as a way to curb *Before LUC (1-2)* scenarios [3,23,24,28,29,30]. Hence, *Before LUC (1-2)* scenarios were already known before the introduction of LUC [28,29,30,33,34,35,36,78,79,80]. A fundamental weakness of *After LUC (3-5)* is that it focuses on food quantity at the national level (as a single aspect of food security) thereby ignoring the aspects of food quality, food availability, food accessibility, and food usage/utilization aspects at the household level. Also, the farmers have the requisite skills for managing climate change situations and do not have the knowledge (and capacities) to deal with natural disasters. These can, in turn, be considered as the main factor of its failure to improve the status of food security after its introduction. It is important to note that the *Before LUC (1-2)* scenarios had food security problems. A major difference between it and the *After LUC (3-5*) scenarios is that multiculture enabled food diversification with low quantity of food production in the *Before LUC (1-2)* whereas monoculture is currently enabling high quantity of food production with low food diversification in *After LUC (3-5*). 

Since the findings show that there were scenarios of food insecurity in both periods (before and after LUC) under two different conditions of Multiculture (Before) and Monoculture (After), the authors argue that LUC cannot be considered as the main driver of food insecurity in the area. Rather, combined with other external factors like climate change, market imperfections, knowledge gaps, it has failed to revert the existing Multiculture-based form of food insecurity and contributed to a new Monoculture based form of food security. Therefore, this raises the question of how to deal with these two extreme scenarios of food insecurity in a responsive way. The authors suggest that the best approach would be finding strategies which could keep both situations (multiculture and monoculture) under different conditions as an optimum solution to this problem. Drawing from the study of Ntihinyurwa et al. [8] about the positive impacts of land fragmentation in Rwanda, we suggest that market oriented and monoculture based land use consolidation be applied in more homogenous areas with less variability in agro-ecological, physical (soil, slope, water, etc.), socio-economic and climatic conditions, and keep the multiculture in more heterogeneous conditions as a risk management strategy, climate change resilient and adaptation strategy to land fragmentation and food insecurity problems in Rwanda, as stipulated by the SDGs (2,13 and 15) in the Agenda 2030.

**Figure 7:**
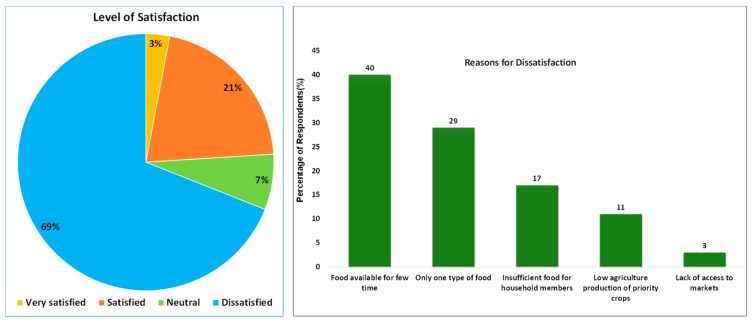
Level of farmers’ satisfaction about LUC and reasons behind it.

Furthermore, the explanation for this failure is also shown by the level of farmers’ satisfaction with LUC and the reasons for their satisfaction as shown in Figure 7.

The level of farmers’ satisfaction about LUC is based on pre-established responses. However, the reasons behind their dissatisfaction is not based on pre-established responses, but rather on unguided feedback received from the respondents through follow-up questions. The majority of respondents report being unsatisfied with LUC, accusing it to negatively affect the sustainable/stable availability of balanced food sufficient for their household members (69% and 86% respectively). The majority of the critical informants have also blamed the lack of access to the food markets (market imperfections), leadership problems, low education level of farmers (food usage education) and natural disaster (including drought and climate change) as some other factors that led to LUC failure to meet food security needs of local farmers. However, the key informants note that the introduction of Kitchen Gardens (*Utulima twigikoni*) as new governmental strategies to accompany LUC in its process of meeting food security targets at the household level. In this program, households are taught to make small gardens around their residencies where they can grow small scale vegetables which could help them in their daily life to meet the balanced dietary needs, even though their production is still at the lowest level.

### 4.3. Gap between LUC Principles and Their Implementation

#### 4.3.1. LUC Objectives and Guiding Principles 

The LUC was initiated in Rwanda in 2008 as the central pillar of CIP initiated earlier by the Ministry of Agriculture and Animal Resources (MINAGRI) in September 2007 with a goal to increase agricultural productivity of high-potential food crops and to provide Rwanda with greater food security and self-sufficiency. According to the *Ministerial Order n°14/11.30 of 21/12/2010* of Rwanda [23] which deals with land consolidation models in Rwanda, the ultimate goal/aim of LUC is the rural development and promoting agricultural transformation that increases agricultural production and improves the lives of Rwanda´s people in rural areas. The *Article 5 of the Ministerial order n°14/11.30 of 21/12/2010* [24] defines three models of land consolidation for farm productivity purposes which include: the use of facilitated farming contract; cooperative farming; and farming corporation. Furthermore, Article 14 of the *Ministerial order n°14/11.30 of 21/12/2010 of* Rwanda [26] stipulates that the designing of land use consolidation and its implementation shall respect the following guiding principles:

- Not only the improvement of agricultural production but also the improvement of rural livelihoods;
- To ensure that identified potential land use is market-oriented;
- To determine possibilities of encouraging farmers and private investors to voluntary participate in the project and to support their participation.
- To ensure that women, youth and members of vulnerable groups participate in land consolidation project with the intention of promoting its practical use and to ensure optimum productivity and benefit to them;
- To support any existing off-farm employment opportunities to support the farm laborers that may lose employment due to land consolidation with the intention of promoting its practical use and to ensure optimum productivity;
- To aim at attracting investors who are practically committed;
- To apply democratic principles, use of consultative methods on any issue to be tackled and provide an avenue for members of the community to express their comments on various programs.

The *Official Gazette no 52 of 27/12/2012* of Rwanda [23,24] provides that the selection of an appropriate land (use) consolidation model (with the intention of promoting practical use and ensuring optimum productivity relevant to each particular location) shall be a result of collaboration between the Ministry in charge of agriculture, landowners, land tenants and other stakeholders concerned with the identified local farming areas. The farmer may pay for the inputs at the time of purchase or after harvest, using the proceeds of the sale of the crop. 

Based on all of the above information, it is inferable that the LUC objectives, models and principles support food security improvements, but at the national level. Therefore, the inability of LUC to improve food security implies that there are gaps between LUC principles and its implementation. 

#### 4.3.2. LUC Process Is Neither Participatory, Tenure Security Responsive, nor Food Security Responsive

A starting point for gaining an insight into the situation of land-use planning (in this aspect, land use consolidation) and food security was to know whether land tenure is secure enough for the rural people of Nyange. Despite that the land in Nyange is formalized, the participants in the LUC believed that their tenure is not “secure enough” to the level that ensures *“participation in economic development”* and *“protection from the loss of full or partial land access or land rights,”* as some of the farmers mentioned. They noted that LUC diffuses their right to farm in their preferred manner which could have helped them to meet their dietary needs and food preferences. One of the farmers advanced that the approach of LUC demands that:

“Farmers must forego their traditional intercropping ways of farming and commit to adopting the program’s use of single cultivation of priority crops in order. This makes it difficult to produce for household dietary satisfaction, market needs”.

By using the word *“forego”* in expressing their situation, it indicates a lack of a major land-use right which the farmers consider deterrence in their quest to become food secure. Concerning their tenure security situations, another farmer echoed their collective viewpoint, saying:

“We do not have the right to cultivate the crop of our choice. We do not have the right to choose the priority crop of the adjacent sites, and we are not allowed to cultivate crops not considered as priority crops by the government. If we ignore these rules, we are condemned to pay some penalty fees between 10 to 50 thousand Rwandan Francs based on the size of land cultivated with non-priority and non-chosen priority crops”.

The above statement indicates that the farmers do not have tenure security on their land. It also means that they cannot decide on *cultivating what they need most (and best) for their household food needs.* As another farmer put it, *“we cannot afford to decide on what we farm and this situation affects what we eat since not all of us can afford to buy other foodstuffs from the market for us to have diverse food items at home.”* The experience expressed by the farmers is indicative of insecurity in the participation of farmers in the program in ways they deem suitable for improving their food security needs. The respondents who participated in LUC gained access to improved agricultural seeds and fertilizers but not necessarily the seeds of their particular needs for household food security improvement. This is because LUC is an “imposed innovation” [81]. As a consequence, food insecurity remains a challenge at the household level. Although different studies reiterate the presence of high security of tenure (land ownership rights) in Rwanda after the national systematic land registration program which left 11.4 million parcels demarcated (more than 90% of the national coverage), and 7.2 million land titles issued [82], these findings from different data sources (key informants, landowners/farmers, and the literature/different reports) question the issue of enjoyment of the use rights over land. This study considers tenure security to mean not only the security of ownership rights, but also the enjoyment of the use rights with freedom—freedom of making decisions about the use of their lands in the way which meets their dietary needs and food preferences. Similar findings were reported by Lengoiboni et al. [83] in their study about the impacts of women land rights registration on household food security in Rwanda. They have mentioned that despite the tenure security of their land rights after the land registration program, women faced tenure insecurity when it comes to the limitations or land use restrictions in the formal land law through the crop intensification program and LUC, by giving them little room for freedom to grow food crops of their choice, thereby negatively impacting their food security at the household level.

**Figure 8:**
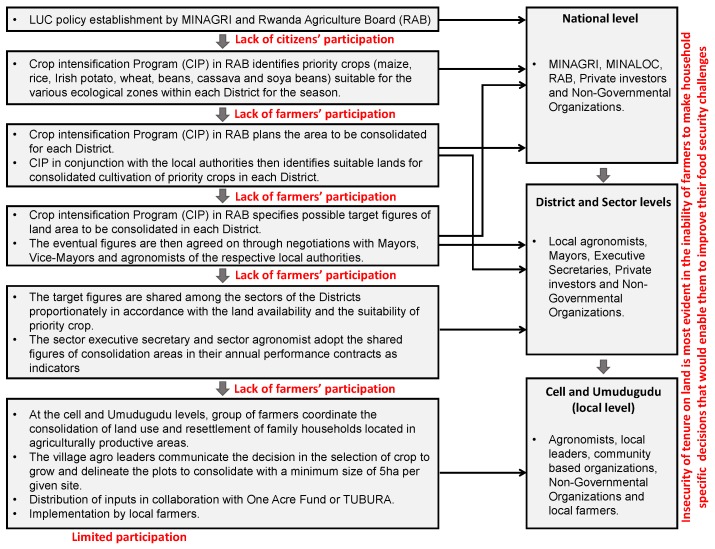
The overall process of the LUC.

The field study and the review of the existing documents have shown the main stakeholders of LUC to include the Ministry of Agriculture and Animal Resources (MINAGRI), Rwanda Agriculture Board (RAB), Ministry of Local Government (MINALOC). Local authorities (at the district, sector, cell and village levels), NGOs, local farmers, private investors and community based associations/organizations are also involved in LUC process as stakeholders as shown in Figure 8.

From the process of LUC represented in Figure 5, it can be seen that local people only implement the instructions of local authorities in a top-down way. The evidence from local authorities reported the problem of performance contracts and targeted figures to meet, as one of the factors inducing them to force local people to grow the government approved priority crops chosen for them at national and district level based on different agro-ecological zones in order to protect their jobs. A significant feature of the process of the LUC is that it lacks citizens’ participation in its core areas for instance, in its establishment, crop intensification decisions, crop types to be used, land sizes to be cultivated, land areas to be used and specific activities to be indulged. Despite that land tenure has been regularized in Rwanda, land rights (related to decisions on the *what*-and-*how* aspects of land uses) that would enable them to solve their household specific food security challenges are either unprotected or infringed upon by the LUC process. This is evidence that the farmers involved in the LUC are not secure enough in ways that would secure food for their families. Hence, any efforts towards improving food security must include the widening of the margins of tenure security on land.

These findings are in accordance with the findings of Huggins [34], Kathiresan [35], Konguka [78], and Kathiresan [79] who managed to show the top-down implementation procedure without negotiations with local people and the pressure of local authorities on local people to participate in order to meet their performance contracts. The chosen priority crops in Nyange Sector are Maize, Irish Potatoes, Beans and Wheat respectively.

**Figure 9:**
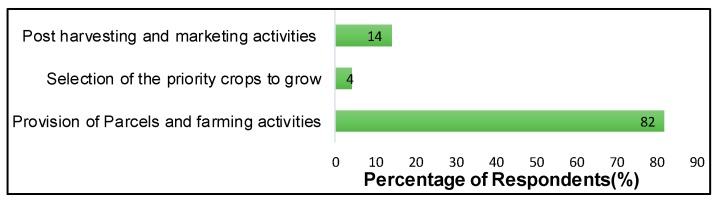
Level of farmers’ participating in LUC (based on the household survey).

Emanating from the process of LUC is the issue of farmers’ participation. The information from the household survey (see Figure 9) reveals a limited level of farmer’s participation in the decision-making process, particularly in the selection of priority crops. 

A majority of respondents declare their participation to be only limited to farming activities of the pre-chosen priority crops. This makes the food security component of the LUC implementation a top-down process (with no farmers’ involvement in decision making about the selection of priority crops suitable to their local area). Also, citizens were not involved in the formulation of the project. These imply that some fundamental guiding principles of the LUC are not being followed. These principles include improvement of rural livelihoods, voluntary participation based on democratic and consultative methods as the basis for its bottom-up implementation. These confirm the gap in the LUC implementation, which is why it has not been able to improve food security challenges in Nyange Sector.

## 5. Making Rwanda’s Land Use Consolidation to Generate Food Security: Why Tenure Responsive Land-Use Planning Matters?

The implementation of LUC in Nyange Sector of Rwanda is indicative of why participatory and tenure securing approach to land-use planning is necessary in order to ensure inclusive decision making in land-use and consolidation practices. This is why adopting a tenure responsive land-use planning practice is necessary for making LUC responsive to food security. However, in adopting a tenure responsive land-use planning perspective, it is necessary to ensure that the following critical issues are considered in any operational approach to improving food security:

- Food security focused principles and objectives to be maintained and principles slightly modified: LUC objectives are food security oriented, but the principles seem to focus on improving food quantity at national level, thereby ignoring other aspects like food quality, food accessibility, food sustainability and food utilization as prerequisite of food security at household level. The implication of this is that any approach to improve tenure security must retain existing LUC objectives but improve the principles to make them oriented towards food security at the household level in all its aspects.
- Land ownership: The CIP works through LUC, through the participation of farmers who volunteer to consolidate aspects of their farm operations (while retaining individual ownership of their land) to improve their household food security. This aspect ensures tenure security and has to be retained in any new approach.
- Participation in all food security sensitive aspects of LUC: Although the LUC program is voluntary, participation in the program is quite imposed in many cases. There was a limited role of citizens and farmers because the farmers only had to provide their land parcels and then farm the consolidated plots with no involvement in the aspects of crucial decisions about food security issues. The type of participation observed at the local level has been referred to as “forced and compulsory involvement of local people in the LUC policy done by the local authorities on the pressure to meet their target figures committed in their annual performance contracts with the government” [24]. There is a need to improve participation and inclusiveness in the LUC. Tenure responsive land-use planning provides opportunities for participation and stakeholder engagements in land-related activities.
- Crops selection that is capable of bridging food security gaps: Farmers’ commitments to participate in the LUC is on the condition that they abandon their usual intercropping cultivation techniques in favor of cultivating only a single government-approved crop. There is a need to widen the margins of exercise of property rights by farmers to enable them to engage in broader crop selections. Tenure responsive land-use planning enables an adequate understanding of land suitability issues, as well as agricultural decisions related to crops.
- Land tenure decisions that are responsive to food security: Land tenure security is a challenge when farmers commit to LUC conditions because they lose their rights to making household specific food security generating decisions. There is a need to widen the margins of exercising property rights by farmers to enable them to engage in broader decisions based on their specific household food situations and experiences. LUC is based on a generalized Agro-Ecological Zones that do not consider the conditions of local soil (which is usually best known to local farmers). Apart from this current study, many other studies have argued that LUC has had an adverse effect on individual land use rights [8,26,35,36,80,81].
- Household food security focused outcomes: Respondents participating in LUC commonly reported increased crop availability and quantity of the priority crops but confirmed that food insecurity remains a household challenge. This is due to the silos focus of LUC only on boosting food quantity and availability at the national level, ignoring the aspects of quality, accessibility, and sustainability at the household level. Any new approach must embrace the focus on food security improvement in all of its aspects in both principle and practice at the household level.
- External risks caused by natural disaster and market risks: Respondents also reported that their LUC operations face land related risks such as climate change leading to the change in rainfall patterns, floods, drought, and high food prices. Land-use planning is necessary to ensure mitigation against climate change and natural disasters.
- Internal risks caused by knowledge capacity: There is the challenge of poor knowledge capacity on food security issues –such as low awareness of nutrition, food (or foodstuff) combination to boost balanced nutritional diet. Capacity development is essential for equipping participants with the requisite knowledge on how to deal with market imperfections, nutrition education, and skills necessary for improving and sustaining food security at the household level.
- Market perfections improvements: As LUC has been revealed to be associated with the reduction in agriculture production of priority crops and the increase of their prices on the market, which makes them unaffordable to the poor farmers, and hence their inaccessibility for foodstuffs diversification and nutritional balance purpose, there is a need to create better food market conditions in the new approach. This issue has been raised by many other authors such as Habyarimana and Nkurunziza [84] who have shown how LUC success to meet its food security targets is highly dependent on the level of market perfection as a high market reliant program. Without very well-functioning food market allowing all the participants to afford other foodstuffs besides their production from their priority crops, LUC will never achieve its food security improvement objectives.

**Figure 10:**
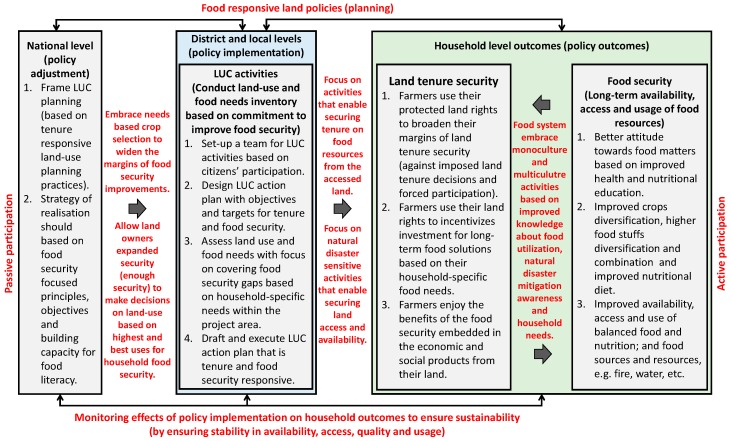
The operational framework for ensuring food security responsive LUC (based on tenure responsive land-use planning).

Based on the above-listed conditions (which are vital learning points deducible from the case study), this study recommends a renewed LUC process that is framed around land tenure security cum food security improvements. Hence, the need to adopt a tenure responsive land-use planning based approach, which is appropriate for improving the Rwandan situation because it is a process that links land-use, tenure, consolidation issues to food security improvements (Figure 10). 

Any operational approach for LUC to improve food security (through tenure responsive land-use planning), should at the least have four core components of action at national, district and local levels: (1) The aspect of policy (to put in place and enable food responsive land policies that will determine the planning of actions for food security). (2) The aspect of citizens’ participation (to ensure that households appropriately undertake food security related decisions). (3) The implementation aspect (to ensure that appropriate activities are carried out with the objective of aligning land-use decisions towards food security objectives). (4) The Monitoring (and evaluation) aspect (to ensure that food security outcomes are sustainable for continuity in improvement). These four aspects are further explained in details.

A starting point for improving food security would be to ensure that the policy aspect is based on land policies that recognize the need to improve food security (food responsive land policies) to ensure that planning induces elements capable of causing food security. Such policies should be made at the national level and translated through the district to the local level to provide the framework for the development of food security in all its aspects of food availability, accessibility, food quantity, and food utilization food quality and food sustainability.

A land policy that is designed to be responsive to land tenure security and food security can provide the implementation pathway for reframing of LUC (based on tenure responsive land-use planning practices). It can provide the strategies and principles, objectives and capacity building measures (for food literacy) for producing food security outcomes. For this to happen, such a policy has to be translated into a district and local level implementation, LUC activities should combine land-use, tenure security, and food security activities by embracing needs-based crop selection necessary for widening the margins of food security improvements at the household level. Policy implementations should also allow landowners to have broader land tenure security (enough security) to make decisions on land-uses (based on highest and best uses) for household food security. With these in place, implementation activities should include conducting land-use, and food needs inventory (with a commitment to improving food security) by fulfilling four actions. First, it will be necessary to set up a team for LUC implementation that is based on citizens’ participation. Second, it is vital to design LUC implementation (or action plan) based on objectives and targets for tenure and food security. Third, assess land use and food needs with a focus on covering food security gaps based on household-specific needs within the project area. And, fourth, it is mandatory to draft and execute LUC action plans that are tenure and food security responsive. Focus on activities that enable securing tenure on food resources from the accessed land. It is important to mention the nurturing relationship which land tenure and food security share –improved land tenure security usually results in improved food security, and vice versa. These are possible when the participation of farmers (or landowners) focus on implementing natural disaster sensitive and climate resilient activities that can enable securing land access and availability. The outcome at the household level would be improvements in land tenure security which will enable the farmers to use their land rights to incentivizes investment for long-term food solutions based on their household specific food needs.

Most importantly, it will enable them (farmers) to enjoy the benefits of the food security embedded in the economic and social products from their land. The implication of this is that farmers can practice agricultural land-use activities (whether monoculture and multiculutre), based on their household food priorities, and with improved knowledge about food utilization and natural disaster mitigation. The impact would be long-term availability, access, and usage of food resources (food security). Food security will occur due to a better attitude towards food matters based on improved health and nutritional education; improved crops diversification, higher foodstuffs diversification, and combination; and improved nutritional diet in households.

Food security must be made to have a stable effect on household health and nutrition. This demands making it stable such that it becomes a way of living which can benefit all household members. To ensure this, it is necessary to monitor the effects of policy implementation on household outcomes to ensure sustainability in availability, access, quantity, quality and usage of food.

Operationalizing successful activities for ensuring a food security responsive LUC can only be possible through various kinds of participation ranging from passive to active. While it may not be realistic to expect active participation of rural people in national level strategies, inputs from the local levels should be sought in order to understand their correct food security gaps (passive participation). However, activities at the local level should actively engage farmers so that they are empowered to make critical decisions on how to solve their food security problems and thoroughly enjoy their use rights over their lands. For instance, the government should engage local people in decision making on the choice of crops to grow. More consultative approaches should be applied to make sure the local people are well involved in the procedure after a long capacity building campaign, in order to give them the ability to participate. This requires a bottom-up approach in the implementation process instead of top-down impositions. This means limiting the role of government at the local level to the provision of technical and managerial advice and assistance, with the local people freely using their lands (active participation). Participation is not only one of the critical aspects of the operational procedure for attaining food security, but it is also a precondition for achieving food security. Yet, with more than 90% of all its land resources demarcated and registered in the national cadaster, this can be a very important potential for the success of this new approach to Land Use Consolidation in Rwanda, since a presence of a cadaster is a prerequisite and success factor for land use planning.

## 6. Conclusions

It is evident that the reason Rwanda implemented the LUC (within its CIP) to ensure food security, but failed to achieve it at the local level in Nyange sector. This study achieved six key objectives. First, identified the general food insecurity scenarios that resulted from the implementation of LUC in Nyange Sector in Rwanda. Second, it identified the gap between LUC principles and their implementation, leading to its poor performance on food security at the household level. Third, it revealed the farmers’ perceptions on the issue to show that the outcome of LUC did not support household food security. Fourth, it identified the specific scenarios, and used them to explain how and why LUC failed to ensure food security at the local level in Rwanda. Fifth, it also showed that the LUC process was neither participatory nor tenure security responsive, and so failed to be food security responsive. Sixth (and finally), it provided a renewed perspective for improving the food security situation by adopting a tenure responsive approach to the implementation of LUC at the local level.

It is not uncommon to use the analysis of agriculture policies as a pivotal part of assessing food security. In this study, the LUC (which is the cornerstone of the CIP) is a central part of Rwanda’s National Agricultural Policy [85]. The Rwandan National Agriculture Policy recognizes LUC (through CIP) as an instrument for enhancing the “availability, accessibility and optimal use of good quality seeds enhance crop yields and their subsequent contribution to food security, balanced nutrition, value of the product in the market, and economic growth” [85]. This is why this study has focused on the LUC, rather than on the wider agriculture policy of Rwanda. It is also logical to assume that the land policy of Rwanda has a special role in the food security improvement of the country. This study acknowledges that a land policy can set the enabling framework for securing tenure, which has been found to have a direct link to food security. The Rwandan Government [86], in recognition of aspects of its current land policy to ensure full security of tenure on the use of land by its citizens, have initiated a new draft land policy. The draft policy on land is already in place and consultations are underway to come up with a binding document to replace the existing policy which has been in force since 2004 [87]. All of these efforts are indicative that government recognizes that additional efforts are needed to truly make food security a reality.

These policy efforts are necessary as they set up an enabling environment for the operationalization of strategies (or approaches) that can produce food security outcomes. In this regard, this study makes two main contributions to knowledge, each to literature and practice. First, it contributes to planning literature by introducing the concept of tenure responsive land-use planning as a method for food security improvement. By doing this, it demonstrated that tenure responsive land-use planning matters in food security issues because it connects land-use decisions to food security outcomes. Second, it contributes to the how-to aspect (practice) by presenting a framework for operationalizing tenure responsive land-use planning to respond to food security improvements. Furthermore, it confirms previous findings that indicate that Rwanda’s LUC is unresponsive to food security [8,26,35,36,80,81]. It also evokes another dimension of land tenure insecurity which has been ignored by other researchers—that is, the capacity for landowners to make critical decisions that affect their food security status. A critical issue emerging from the study is that, the imposition of priority crops on farmers and the denial of farmers to have a say on what crops are planted in their farms or farms adjacent to their farms constitutes tenure insecurity in the context of land-use. Land tenure security can be the legal and social freedom to make decisions on what, where and how to use land (and crops) to achieve household food security objectives. When this kind of freedom is denied, as seen in Rwanda, it affects the production of food crops in accordance to specific household needs. The functionality, efficiency and sustainability of farmland environments, provides farmers with the challenge to innovate and secure food in quantitative and qualitative manner, as well as to sustain production. This is because appropriate land-use and tenure security are capable of inducing food security [1,7,8,10,11,12,13,14,15,16,17,18,19,20,21,22,23,24,25,26,30,31,32,33,34,35,36,37,39,40,41,42,43,44,45,46,47,48,49,50,51,52,53,54,78,79,80,81,82,83,84,85,86,87].

Finally, the data presented in this study are not peculiar to Rwanda alone. Most countries of the Global South (especially in sub-Saharan Africa) share some of the experiences identified in this study –a situation where food insecurity is exacerbated due to lack of tenure security on agricultural land-use decisions. Apart from the operational framework for improving food security (through tenure responsive land-use planning), there is one central lesson that can be drawn by countries where local communities are food insecure. That lesson is that land tenure security, in the context of food security, goes beyond access and use of land. It entails making household specific decisions on land-uses with the purpose of improving food security situations.
